# Effect of breed and diet on the *M. longissimus thoracis et lumborum* transcriptome of steers divergent for residual feed intake

**DOI:** 10.1038/s41598-023-35661-z

**Published:** 2023-06-03

**Authors:** Kate Keogh, Clare McKenna, Sinead M. Waters, Richard K. Porter, Claire Fitzsimons, Mark McGee, David A. Kenny

**Affiliations:** 1 Animal and Bioscience Research Department, Teagasc Grange, Dunsany, Co. Meath, C15 PW93 Ireland; 2 School of Biochemistry and Immunology, Trinity College Dublin, Dublin 2, D02 R590 Ireland; 3 Livestock Systems Research Department, Teagasc, Grange, Dunsany, Co. Meath, C15 PW93 Ireland

**Keywords:** Transcriptomics, RNA

## Abstract

Improving cattle feed efficiency through selection of residual feed intake (RFI) is a widely accepted approach to sustainable beef production. A greater understanding of the molecular control of RFI in various breeds offered contrasting diets is necessary for the accurate identification of feed efficient animals and will underpin accelerated genetic improvement of the trait. The aim of this study was to determine genes and biological processes contributing to RFI across varying breed type and dietary sources in skeletal muscle tissue. Residual feed intake was calculated in Charolais and Holstein–Friesian steers across multiple dietary phases (phase-1: high concentrate (growing-phase); phase-2: zero-grazed grass (growing-phase); phase-3: high concentrate (finishing-phase). Steers divergent for RFI within each breed and dietary phase were selected for muscle biopsy collection, and muscle samples subsequently subjected to RNAseq analysis. No gene was consistently differentially expressed across the breed and diet types examined. However, pathway analysis revealed commonality across breeds and diets for biological processes including fatty acid metabolism, immune function, energy production and muscle growth. Overall, the lack of commonality of individual genes towards variation in RFI both within the current study and compared to the published literature, suggests other genomic features warrant further evaluation in relation to RFI.

## Introduction

Feed accounts for up to 75% of the variable costs in beef cattle production systems^1^ thus having a fundamental influence on the economic sustainability of the enterprise. Improving feed efficiency has the potential to reduce feed costs while maintaining economically favourable traits such as growth and muscle gain^2^. Residual feed intake is a measure of feed efficiency defined as the difference between an animal’s actual versus its predicted feed intake^3^. Animals with low residual feed intake (RFI; efficient) status typically consume between 10 and 20% less feed than their high-RFI (inefficient) counterparts for the same level of performance^4–9^. RFI, which has become the trait of favour for balanced identification of feed efficient animals is an attractive trait for selection in breeding programmes due to its moderate heritability^10–13^, and the weight of published evidence indicates that the trait is not, in the main, antagonistically correlated with other economically relevant traits at either a genetic or phenotypic level^13,14^.

However, the main impediment to sustained genetic progress and adoption of selection for feed efficiency is the logistics and expense of measuring individual animal intake necessary to accurately identify feed efficient animals. Consequently, the development of predictive biological markers that can be used to accurately select cattle with improved feed efficiency potential, is an attractive alternative to direct measurement^14^. An additional concern for forage based beef production systems, such as that practiced in Ireland and many temperate regions throughout the world, is the potential for a genotype x environment interaction manifested as inconsistent feed efficiency and animal performance ranking across different diet types. For example, in most regions worldwide, prime cattle are reared at pasture up to weaning. However, there is significant variation thereafter in backgrounding and finishing regimen depending on the relative cost of forage and concentrate feeds at the target age at slaughter. Variation in energy density and intake capacity characteristics of feedstuff also impacts feed efficiency potential. Moreover, although relatively repeatable, we and others have reported re-ranking of cattle for RFI when offered either the same diet^4^ or diets differing in chemical composition/energy density^15^ over successive feed intake recording periods^5,15^. This is strongly indicative of a genotype x environment interaction for feed efficiency. Indeed, breeding values for feed efficiency within the national breeding programme in Ireland and other countries are typically derived from animals consuming a high-energy diet, however, the vast majority of commercially produced cattle in Ireland are produced on grass-based diets. Furthermore, it is well established that different breeds have different inherent feed efficiency capacity^1^. In light of this, research to elucidate if a genotype x environment interaction exists is warranted in order to inform future breeding programmes^2^.

High-throughput genomic technologies offer the tools to elucidate the genetic architecture of economically important traits such as RFI with a view to informing industry deployment of DNA based biomarkers. It follows that genomic investigations of this complex trait should include the influence of environmental factors such as diet and breed. Transcriptomic analyses can provide evidence to focus on the critical genes and regulatory elements likely to affect a phenotype. Thus, genes consistently differentially expressed across diet and breed with regard to RFI are likely to be robust candidates for further interrogation or indeed biomarker selection. To date there has been little to no agreement between studies that have utilised global transcriptomic or GWAS approaches in terms of identifying common genomic regions associated with variation in RFI, particularly across contrasting breeds and, or, dietary regimens^1,14,16^. Therefore, further information is required on the influence of a genotype x environment interaction on differential gene expression profiles for the RFI trait.

Skeletal muscle, while a tissue of obvious economic importance to beef cattle production, is a highly metabolically active organ accounting for up to 50% of body mass of cattle^17^. In addition, different cattle breeds differ in skeletal muscle gene expression profiles^18^, therefore, it is logical that variation in the metabolism of this organ could influence feed efficiency status. Thus, the aim of this study was to characterise the global transcriptome of skeletal muscle tissue in order to elucidate the molecular mechanisms underpinning RFI in muscle of beef cattle and whether this is consistent across (i) contrasting breeds and (ii) dietary regimes. Such information could be harnessed, to select key genes that could be further interrogated for the presence of genetic variants that may regulate the expression of RFI in beef cattle. Such information, following appropriate validation, should facilitate the selection of animals with enhanced feed efficiency potential and is implementable through genomic selection breeding programmes.

## Results

### Animal model

This experiment was conducted as part of larger study designed to examine the within-animal repeatability of intake, growth and feed efficiency between the growing and finishing stages of the lifespan of Charolais (CH) and Holstein Friesian (HF) beef steers offered contrasting diets over consecutive test periods^19,20^. Descriptive statistics based on dietary intake, RFI and growth rates are presented separately for both breed types across the three dietary phases in Table 1. For both breed types, in all three phases, mean RFI values were, by design, close to zero. The effect of RFI status and diet on dry matter intake (DMI), RFI and performance is presented in Table 1. In the first high concentrate phase (H1), high RFI (H-RFI) CH steers consumed 16% more, and H-RFI HF steers consumed 12% more than their low RFI (L-RFI) counterparts, respectively (*P* < 0.001). In the zero-grazed grass phase (ZG), H-RFI CH steers consumed 8% more, and H-RFI HF steers consumed 11% more, than their L-RFI counterparts, respectively (*P* < 0.001). In the final high concentrate phase (H2), H-RFI CH steers consumed 15% more, and H-RFI HF steers consumed 17% more than their L-RFI counterparts (*P* < 0.001). In each dietary phase within breed, mid-test metabolic body-weight (MBW) and average daily gain (ADG) did not differ (*P* > 0.05) between the two contrasting RFI groups. All steers used in this study were ranked into terciles for RFI groups for each breed type, resulting in 30 and 25 per Low-, Mid- and High-RFI terciles for CH and HF breeds, respectively. From each of the High and Low-RFI groups for each breed and diet type the 12 most extreme for efficiency and inefficiency were selected for tissue sampling and inclusion in this study. Consequently, due to the difference in number between RFI group and those extreme animals subsequently selected for RNAseq, not all the same animals were used for tissue sampling across the consecutive diets. Specifically for the CH breed, 2 steers remained L-RFI between H1 and ZG diets, 3 remained L-RFI between ZG and H2 diets, with 2 steers consistently selected as L-RFI between the two HC diets. For the H-RFI CH steers, 1 steer remained H-RFI across the 3 dietary phases. Three steers remained H-RFI between H1 and ZG diets, whilst a separate group of 3 steers remained H-RFI between the ZG and H2 diets. Between the two high concentrate diets, 4 CH steers remained H-RFI. For the HF group, no steer remained either H- or L-RFI across the three diets. For the L-RFI groups, 2 and 1 HF steers remained L-RFI between H1-ZG and ZG-H2 diets, respectively and 3 steers were consistently designated as L-RFI across the two high concentrate dietary phases. Similarly for the HF steers, 3 steers were H-RFI across the two high concentrate diets, whilst 2 and 1 steers were consistently H-RFI between H1-ZG and ZG-H2 dietary phases, respectively.

**Table 1:** Feed intake, RFI and growth traits for CH and HF steers ranked low and H-RFI offered different diets across their lifecycles.

Trait	Breed	H1				ZG				H2			
		Low	High	s.e	P-value	Low	High	s.e	P-value	Low	High	s.e	P-value
DMI (kg/d)	CH	7.8	9	0.11	< 0.001	8.8	9.4	0.08	< 0.001	10.8	12.3	0.16	< 0.001
	HF	8.3	9.3	0.15	< 0.001	9.1	10	0.11	< 0.001	11.6	13.6	0.2	< 0.001
RFI (kg/d)	CH	− 0.5	0.56	0.04	< 0.001	− 0.35	0.35	0.026	< 0.001	− 0.75	0.76	0.058	< 0.001
	HF	− 0.5	0.53	0.047	< 0.001	− 0.42	0.56	0.048	< 0.001	− 1.01	1.03	0.086	< 0.001
ADG (kg/d)	CH	1.3	1.4	0.05	0.43	1.4	1.4	0.03	0.96	1.4	1.4	0.05	0.64
	HF	1.4	1.4	0.04	0.78	1.2	1.3	0.04	0.85	1.3	1.3	0.07	0.98
MBW (kg/d)	CH	95	96	1.1	0.79	96	97	1.1	0.8	96	97	1.1	0.79
	HF	81	80	1.7	0.9	82	80	1.7	0.9	82	81	1.5	0.9

*DMI* dry matter intake, *RFI* residual feed intake, *ADG* average daily gain, *MBW* metabolic body weight, *CH* Charolais, *HF* holstein Friesan, *Low* low RFI, *High* high RFI, *s.e* standard error of the mean. *H1* high concentrate, phase 1, *ZG* Zero grazed grass, phase 2, *H2* high concentrate, phase 3.

### RNA-seq analysis

RNA sequencing reads were successfully generated for all samples, with approximately 22 million reads per sample on average (range: 19–25 million). Following alignment of sequencing reads to the bovine reference genome (ARS-UCD1.2), 90.62% of reads on average were identified as mapped. Following filtering of lowly expressed genes in EdgeR, the total number of genes expressed in each comparison was as follows: CH-H1: 10994; CH-ZG: 10190; CH-H2: 10531; HF-H1: 11211; HF-ZG: 11033; HF-H2: 10285. A total of 230 genes were identified as differentially expressed (adj. P-value 0.1; fold change > 1.5) between L- and H-RFI steers across the six comparisons undertaken, comprised as follows: CH-H1: 48; CH-ZG: 19; CH-H2: 28; HF-H1: 44; HF-ZG: 87; HF-H2: 4. Genes differentially expressed in each comparison are included within Supplementary Table 1. No individual gene was found to be commonly differentially expressed across all six breed and diet type comparisons examined. In addition to an evaluation of genes differentially expressed between cattle divergent for RFI in each breed across each dietary phase, the interactive effect between diet and breed was also determined. From this analysis 23 genes were identified as significant based on the interaction of diet and breed, these genes are listed in Supplementary Table 1g.

### Pathway analysis

**Figure 1:**
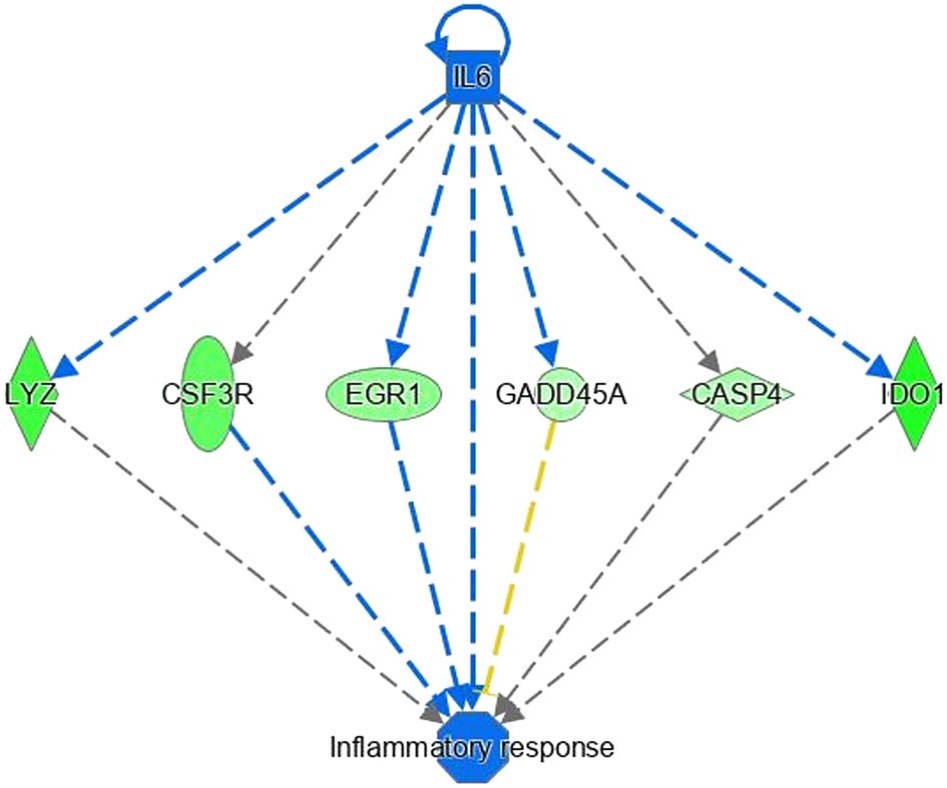
Regulatory effects of IL6 on differentially expressed genes involved in inflammation within the HF-ZG comparison. Colour intensity indicates the expression of genes; with green representing down-regulation in L-RFI steers compared to H-RFI steers.

**Figure 2:**
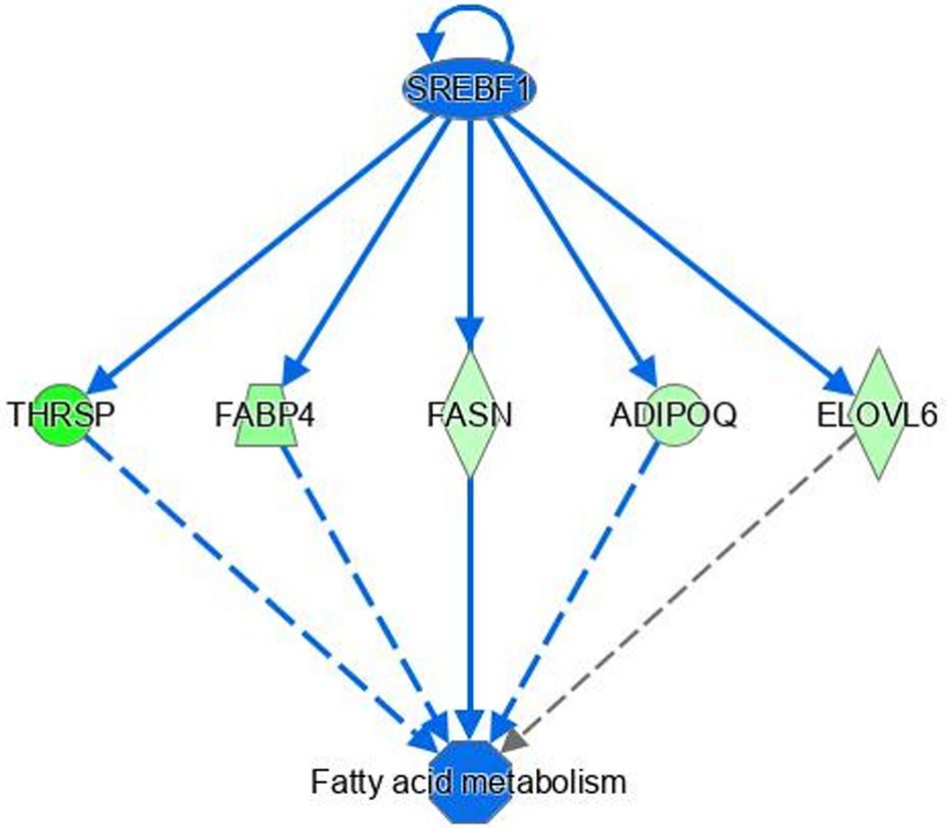
Regulatory effects of SREBF1 genes on differentially expressed genes involved in fatty acid metabolism within the CH-H2 comparison. Colour intensity indicates the expression of genes; with green representing down-regulation in L-RFI steers compared to H-RFI steers.

Pathway analysis of differentially expressed genes revealed enrichment for pathways and biological processes related to fatty acid metabolism, immune function, energy production and skeletal muscle growth. This was apparent through enrichment of these biological processes through functional enrichment analysis in IPA, as well as through enrichment of specific biological pathways. For example, immune signalling pathways such as acute phase response signalling and coagulation system were both enriched immune pathways within the CH-H1, CH-H2, HF-H1 comparisons. Moreover, within the HF-ZG comparison, IL6 was predicted to have a regulatory function contributing to the differential expression of genes involved in inflammation (Fig. 1). Fatty acid biosynthesis and fatty acid activation were enriched within CH-H2 and HF-ZG comparisons, indeed within the CH-H2 comparison, *SREBF1* was predicted to have a regulatory role towards the expression of genes involved in fatty acid metabolism (Fig. 2). Enrichment for mitochondrial related pathways was also apparent within HF-H1 and CH-H2 comparisons. Enriched pathways for each comparison are outlined in full in Supplementary Table 2, with enriched biological processes/functions presented in Supplementary Table 3. As mentioned above a comparison of genes differentially expressed across each analysis revealed enrichment of processes related to fatty acid metabolism, immune function, energy production and skeletal muscle growth. Genes differentially expressed and specifically involved in these processes are presented in Tables 2, 3, 4 and 5, respectively.

**Table 2:** Differentially expressed genes involved in fatty acid metabolism.

Breed and diet	Symbol	Fold change	FDR
CH-H1	*CIDEC*	− 2.076	0.0605
HF-H1	*CIDEC*	− 2.102	0.0696
	*ANGPTL8*	− 3.394	1.26E−05
	*ELOVL6*	− 2.973	3.32E−05
	*FABP4*	− 2.463	0.000617
	*THRSP*	− 2.787	3.24E−06
	*TRARG1*	− 2.366	0.0164
CH-ZG	*ADIPOQ*	− 5.933	7.98E−11
	*CIDEC*	− 6.169	2.4E−12
	*SCD*	− 2.663	0.000174
	*TRARG1*	− 4.656	3.67E−08
	*FABP4*	− 4.564	4.1E−09
	*THRSP*	− 5.716	4.21E−12
HF-ZG	*SLC27A6*	− 2.45	0.0585
CH-H2	*ADIPOQ*	− 3.886	0.0116
	*CIDEC*	− 6.209	7.21E−08
	*ELOVL6*	− 3.564	0.0285
	*FABP4*	− 5.412	2.07E−06
	*FASN*	− 3.018	0.00298
	*THRSP*	− 11.159	1.37E−13
	*TRARG1*	− 4.814	2.98E−05
HF-H2	*PDK4*	− 2.54	0.0941

**Table 3:** Differentially expressed genes involved in immune processes.

Breed and diet	Symbol	Fold change	FDR
HF-ZG	*ARHGAP45*	− 2.849	0.0104
	*CASP4*	− 2.474	0.0863
	*CD3E*	− 2.786	0.0193
	*CD53*	− 3.392	0.00181
	*IDO1*	− 5.369	3.2E−08
	*LCP1*	− 2.806	0.00346
	*PTPRC*	− 3.976	3.96E−05
	*S100A12*	− 3.888	0.000166
	*SAMD9*	− 3.465	0.000803
	*SPN*	− 2.772	0.0234
	*UBD*	− 3.777	0.00027
	*ZAP70*	− 3.713	0.000326
	*ZC3H12A*	2.263	0.0863
CH-H2	*IDO1*	− 3.038	0.0799

**Table 4:** Genes involved in energy production and cellular detoxification differentially expressed between cattle divergent for RFI.

Breed and diet	Symbol	Fold change	FDR
CH-H1	*HPX*	2.043	0.00895
	*NDUFA3*	− 1.916	0.00917
	*NR4A3*	− 2.058	0.015
HF-H1	*CYP4B1*	2.142	0.00391
CH-ZG	*GSTM3*	− 2.519	0.000371
CH-H2	*CKB*	− 3.631	0.00648

**Table 5:** Genes involved in muscle growth differentially expressed between cattle divergent for RFI.

Breed and diet	Symbol	Fold change	FDR
CH-H1	*BTG2*	− 1.813	0.0391
	*CSRP3*	1.751	0.0826
	*EGR1*	2.597	4.3E−07
	*MYH6*	− 3.512	3.69E−11
	*SPX*	1.916	0.0053
HF-H1	*FOS*	2.259	6.41E−05
	*FOSB*	4.486	1.27E−17
	*MYH6*	2.85	3.54E−08
	*MYL6B*	1.894	0.0634
CH-ZG	*MYH4*	2.306	0.022
	*MYH6*	2.773	6.21E−05
HF-H2	*ACTC1*	3.178	0.000166
	*EGR1*	− 2.772	0.00104
	*PAMR1*	2.63	0.0549
CH-H2	*ACTC1*	− 3.161	0.00287
	*EGR1*	3.931	0.000292
	*MYH4*	3.632	0.00476

## Discussion

Understanding the metabolic and physiological mechanisms underlying variation in feed efficiency in cattle is vital for the effective planning of breeding strategies to select the most feed efficient cattle^1,14^. Research examining the biological mechanisms governing RFI and other measures of feed efficiency have mainly focused on young cattle offered high-concentrate diets. In practice, however, most commercial beef production systems worldwide are predominantly forage based^21^ exploiting compensatory growth with animals subsequently finished on a concentrate based diet. RFI is a complex trait that is affected by both genetic and environmental factors with breed and diet having a major influence^4,15^. However, it is not clear from the literature how breed and diet impact the regulation of RFI in cattle. Furthermore, if predictive biomarkers for RFI are to be utilised they must be robust across breed and diet. This study aimed to determine the key regulatory genes and biological pathways differentially expressed in cattle divergent for RFI within two contrasting breeds and across diverse diets with a view to elucidating the key biological mechanisms controlling the expression of RFI in muscle tissue. However, results from this study showed no genes were commonly affected by RFI divergence across the breed and dietary types examined. The lack of commonality in genes identified across contrasting dietary phases in this study may be attributed to the variation in age or stage of development for each breed type during each dietary phase examined. Despite this outcome, results from this study showed commonality in biological processes affected by RFI, including fatty acid metabolism, immune signalling, energy production and muscle growth across contrasting breed type and various dietary sources. The remainder of this discussion will focus on these important biological processes.

### Fatty acid metabolism

A role for genes involved in fatty acid metabolism towards variation in RFI has previously been established across a number of studies utilising diverse breeds and experimental parameters including diet types^22,23^. However despite this, specific key genes regulating such biological processes involved in RFI variation remain to be commonly identified across studies. The same observation was also apparent for the results of the current study where although genes involved in fatty acid and lipid metabolism were identified as differentially expressed within each comparison, no single gene was common across each of the six differential expression evaluations conducted. Despite this, the *CIDEC* gene was down-regulated in L-RFI CH and HF steers in H1 dietary phase as well as in CH steers for ZG and H2 dietary phases. The protein encoded by the *CIDEC* gene binds to lipid droplets and regulates their enlargement, thereby restricting lipolysis and favouring storage^24^. Thus, up-regulation of this gene within the H-RFI steers may indicate greater lipogenesis and lipid storage in the H-RFI cattle, which may contribute to their feed inefficiency, requiring more energy from feed to maintain additional lipid stores within the body. Indeed, this gene was also down-regulated in the liver tissues of the L-RFI CH steers used in the current study following the ZG diet^23^. *CIDEC* expression has been shown to be regulated by insulin, with its expression positively correlated with insulin sensitivity. Similarly *SCD* expression, which was down-regulated in the L-RFI CH steers during ZG dietary phase, has also been shown to be responsive to both systemic glucose and insulin. Indeed *SCD* was also downregulated in pituitary, skeletal muscle, adipose and duodenum tissues of L-RFI Angus steers^25^. Moreover, Mukiibi et al.^22^ reported *SCD* expression as down-regulated in L-RFI steers across three contrasting breed types. Additionally, two further genes, *TRARG1* and *ADIPOQ*, which are both involved in adipose tissue glucose uptake^26^ and thus regulated by insulin sensitivity, were also down-regulated in the L-RFI steers in CH cattle during ZG and H2 dietary phases. Although insulin concentrations were not assessed in the steers used in the current study, nor in that of Mukiibi et al.^22^ and Weber et al.^25^ previous evaluations of RFI in cattle have indicated towards a potential role for insulin and insulin sensitivity towards variation in RFI status, whereby circulating concentrations of insulin were greater in H-RFI cattle compared to their L-RFI contemporaries^14^. Thus, variation in insulin response to clearing circulating glucose may lead to differential expression of genes involved in adipogenesis or fatty acid metabolism and consequently lead to greater energy requirements within the H-RFI cattle.

Additional genes involved in fatty acid metabolism were also down-regulated in the L-RFI cattle across the different breed type and dietary sources used in the current study. These included *ANGPTL8*, *ELOVL6*, *FABP4*, *THRSP*, *SLC27A6*, *ASCM1*, *FASN* and *PDK4*. *ANGPTL8* is involved in the direction of fatty acids towards adipose storage^27^. *ELOVL6* encodes a protein responsible for the rate limiting step in fatty acid elongation^28^ and was previously reported as down-regulated in skeletal muscle, pituitary, adipose and duodenum tissue of L-RFI steers^25^. *THRSP* and *FASN* are involved in the regulation of lipogenesis and fatty acid synthesis, respectively^29^. Indeed Weber et al.^25^ identified expression of both *THRSP* and *FASN* as down-regulated in L-RFI cattle in pituitary, skeletal muscle, and adipose tissues, whilst Alexandre et al.^30^ observed lower expression of *FASN* in liver tissue of L-RFI cattle. Additionally both *ASCM1* and *PDK4* are involved in fatty acid metabolism. The *PDK4* gene, although downregulated in the HF steers in the current study (H2 diet), was previously reported as down-regulated in L-RFI CH steers in the data of Mukiibi et al.^22^. The *FABP4* gene is involved in fatty acid uptake, transport and metabolism. Similarly, *SLC27A6* encodes a fatty acid transporter protein, which was also down-regulated in L-RFI cattle in the liver based transcriptomic study of Chen et al.^31^. Overall, results indicate a greater amount of lipogenesis and adipogenesis in H-RFI cattle which may account for the difference in metabolic requirements. Indeed, pathway analysis predicted a regulatory role for *SREBF1* towards the expression of genes identified as involved in fatty acid metabolism in CH-H2 comparison (Fig. 2). *SREBF1* encodes a transcription factor which is a primary regulator of lipid metabolism^32^. Despite not identifying the same genes across all studies, we have shown consistency across the contrasting dietary regimens utilised in this study along with commonality with previously published results reinforcing the central role of fatty acid metabolism towards variation in RFI in beef cattle. Results from this study also show an involvement of genes related to fatty acid metabolism towards RFI across diverse breed types and varying dietary sources. Indeed, Weber et al.^25^ concluded that the increase in carcass specific gravity of L-RFI cattle suggested a reduced body fat content in the efficient cattle, reinforcing the reduction in fat content in L-RFI cattle.

### Immune response

Similar to genes related to fatty acid metabolism, a role for genes involved in the immune system or immune signalling has been reported previously in studies examining the underlying biology of RFI in cattle^1^. However, unlike the results for the fatty acid genes, which were differentially expressed in each breed and dietary phase evaluated, genes involved in immune system were differentially expressed in CH steers divergent for RFI phenotype under ZG and H2 dietary phases and for ZG dietary phase in HF cattle only. Indeed, with the exception of one gene (*ZC3H12A*) all genes related to immune function were down-regulated in the L-RFI steers in each comparison. This direction of effect is in line with previous studies which also reported an up-regulation of immune related genes in the inefficient H-RFI cattle, across various tissues, breeds and dietary types^22,23^. The differential direction of effect for *ZC3H12A* may be due to the variable response of the expression of this gene dependent on diet, evident through the identification of this gene as significant through the diet and breed interaction analysis. Interestingly, in the current study, the majority of the immune genes identified as differentially expressed were pertaining to differences between H- and L-RFI HF steers during the ZG dietary phase, which may suggest a differential transcriptomic response between varying breed types receiving the same dietary allowance. In particular, genes differentially expressed during the ZG dietary phase for the HF steers, included a number of genes with functions associated with inflammation. Specifically these included *CASP4*, which encodes a protein involved in the inflammasome^33^, as well as genes involved in the regulation of inflammatory processes, including *HBB* and *S100A12*. Additionally *SAMD9*, which plays a role in the inflammation response was also up-regulated in the H-RFI steers. Indeed, some of these genes have previously been implicated towards variation in RFI in cattle. For example Paradis et al.^34^ reported lower expression of *HBB* in the liver tissue of L-RFI crossbred heifers. Moreover, IL6 was predicted to have a regulatory role in relation to the expression of genes involved in inflammation in the HF-ZG comparison, which included the *CASP4* gene. Overall, results from the current study and those from the published literature indicate a role for genes involved in inflammatory processes in contributing to variation in RFI.

In addition to the genes related to inflammation, a number of genes different between HF steers during the ZG dietary phase were associated with T-cell response. T-cells represent an important white blood cell of the immune system, providing a central role in the adaptive immune response. Genes involved in T-cell function differentially expressed in the current study included those directly involved in regulating T-cell response as well as genes involved in T-cell receptor signalling. Indeed similar to inflammation related genes, all genes related to T-cell function were down-regulated in the L-RFI steers. These included *ZAP70*, which plays a role in T-cell development ultimately contributing to the regulation of the adaptive immune response^35^. Additionally, genes involved in T-cell activation were also down-regulated in the L-RFI steers. Specifically these included *SPN*, *UBD*, *ARHGAP45*, *IDO1* and *LCP1*. The protein encoded by *SPN* is involved in the antigen specific activation of T-cells. Whilst *UBD*, *ARHGAP45* and *IDO1* all contribute to T-cell responses as well as modulating T-cell behaviour and immune homeostasis. Indeed, in addition to down-regulation in L-RFI HF steers during ZG phase, *IDO1* was also down-regulated in CH steers during H2 dietary phase. Finally, *LCP1* functions in the activation of T-cell response through antigen co-stimulation. Moreover, genes with functions related to T-cell receptor signalling including *PTPRC*, *CD3E* and *CD53* were also down-regulated in the L-RFI HF cattle during ZG dietary phase. Thus, results from the HF steers suggest an increased adaptive immune response within the ZG dietary phase, however the lack of commonality of these genes with the other diets assessed in the current study as well as the results within the published literature suggest that this result may be specific to the experimental parameters employed.

### Energy production and cellular detoxification

Mitochondria are responsible for 90% of whole body energy production as well as being major regulators of cellular homeostasis through their function as reactive oxygen species regulators^36,37^, thus it is reasonable to expect a potential contribution of such cellular organelles towards variation in feed efficiency. Indeed previous evaluations on the underlying biology governing RFI in beef cattle have indicated towards a role for enhanced mitochondrial function in feed efficient cattle, however, results on this are not conclusive^1,14^. For example, some studies have reported greater expression of genes involved in the electron transport chain and mitochondrial function in L-RFI cattle, whilst other studies identified no differences. Indeed, results on the potential functional differences in mitochondrial function between cattle divergent in RFI are also conflicting. For example while Kolath et al.^38^, Fernandez et al.^39^ and Lancaster et al.^40^ observed increased mitochondrial function in the L-RFI cattle, McKenna et al.^41^ did not observe the same result in liver and muscle tissue of Simmental heifers and bulls. Indeed in their transcriptomic evaluation of skeletal muscle and liver tissues in the same Simmental heifer and bull population, McKenna et al.^41^ observed differential expression of mitochondrial related genes in the skeletal muscle of heifers, with all genes up-regulated in the L-RFI cattle. Thus, the lack of agreement between studies may be due to the use of varying experimental parameters including breed, stage of development and dietary source. Indeed, this was also apparent within the results of the current study, whereby genes involved in the electron transport chain were differentially expressed across breed and dietary source, but in contrasting direction of effect. For example within the CH steers, *NDUFA3*, which encodes a subunit of Complex I of the electron transport chain, was down-regulated in the L-RFI cattle. Conversely though genes involved in complexes of the electron transport chain (*COX3*, *ND3*, *ATP8*, *CYP4B1*) were all up-regulated in the L-RFI HF steers during the same dietary phase (H1), suggesting differential response dependent on breed type. Additionally, genes involved in energy homeostasis including *NR4A3* and *CKB* were both down-regulated in L-RFI CH steers, in H1 and H2 dietary phases, respectively. *NR4A3* encodes a protein involved in regulating energy balance and metabolism, whilst also regulating gene expression that controls oxidative metabolism in skeletal muscle^42^. Indeed, this gene was also identified as affected by both diet and breed in the current study too, evident through the interaction analysis. Similarly, *CKB* encodes an enzyme involved in energy homeostasis, playing a central role in energy transduction in tissues with large fluctuating energy demands such as skeletal muscle. Indeed, the differential direction of effect of these genes is also apparent for *CKB*, which was also observed as down-regulated in the liver tissue of L-RFI CH steers used in this study^23^, but was upregulated L-RFI Nelore steers in the liver transcriptomic study of Tizioto et al.^43^. Thus, results from the current study further reinforce the ambivalence within the published literature on the contribution of mitochondrial efficiency towards variation in RFI in beef cattle.

In addition to the potential role of enhanced mitochondrial function in feed efficient cattle, a role for altered reactive oxygen species production and cellular detoxification was also apparent. Feed efficient cattle have shown lower expression of genes related to oxidative stress as well as lower expression of genes related to cellular detoxification^14^. The same result was observed in the current study where genes involved in cellular protection and detoxification were differentially expressed. These included *HPX*, involved in protecting cells from oxidative stress, which was up-regulated in L-RFI CH steers following H1 dietary phase. Indeed, *HPX* was also differentially expressed in the data of Weber et al.^25^, specifically in that study *HPX* expression was greater in pituitary, liver and duodenum and at the same time lower in muscle and adipose tissues of the L-RFI steers used in that study. Together these results show different responses based on tissue type but also based on breed and dietary source between that of the current study and that of Weber et al.^25^. Additionally *GSTM3* and *GADD45* were both down-regulated in L-RFI cattle during ZG dietary phase in CH and HF steers, respectively. *GSTM3* encodes a glutathione S-transferase involved in detoxification, whilst *GADD45* expression has been shown to be increased in response to cellular stress. Furthermore, *GSTM3* was previously reported as down-regulated in the liver tissue transcriptomic data of Chen et al.^31^ in L-RFI cattle, similar to this current study. Altogether these results show an effect of cellular mitochondrial processing and cellular detoxification towards variation in RFI in beef cattle, which appears to be dependent on the breed type as well as the dietary source employed.

### Muscle growth

Currently data on the contribution of RFI towards variation in skeletal muscle accretion are conflicting with studies presenting positive^4^, negative^13^ or neutral^7,8^ relationships between RFI and ultrasonically scanned *M. longissimus thoracis et lumborum* size. Despite this, results from the current study indicate towards greater potential for skeletal muscle tissue development and growth in the L-RFI steers across the varying breeds and diet types examined. For example, the transcriptional regulatory gene, *EGR1* was up-regulated in CH and HF L-RFI steers in H1 and H2 dietary phases. *EGR1* functions in differentiation through its role as a transcriptional regulator, with the greater expression of this gene within the L-RFI steers indicating greater differentiation and growth in the L-RFI CH and HF steers dependent on the diet type. Indeed, a role for this gene towards greater growth is further evidenced through greater expression in L-RFI cattle in both liver and skeletal muscle tissues^43–45^. Furthermore, following the H1 dietary phase, the L-RFI HF steers displayed greater expression of both *FOSB* and *FOS*, in addition to both of these genes being affected by both diet and breed, evident through the interaction analysis. Both of which were also previously reported as up-regulated in the liver of L-RFI cattle^43^ and both have been shown to have important functions in regulating muscle hypertrophy^46^. Additionally we also identified differential expression of genes involved in cell cycle regulation. In particular, the *BTG2* gene, which has anti-proliferative properties, and is involved in the regulation of the G1/S transition of the cell cycle was down-regulated in the CH L-RFI steers following the H1 dietary phase. Moreover, the *BTG2* gene was also identified as significant in the current study through the interaction between diet and breed. Down-regulation of this gene indicates a continued growth of the muscle tissue compared to the H-RFI CH steers. Indeed, *BTG2* was also previously reported as down-regulated in other studies examining RFI divergence in liver tissue^22^ and skeletal muscle tissue^44^, further suggesting greater growth potential in L-RFI cattle.

In addition to the differential expression of genes with general functions in growth mentioned directly above, we also identified genes with specific functions in muscle development as up-regulated in the L-RFI steers. These included *CSRP3*, *SPX* in the CH steers following H1 dietary phase and *PAMR1*, which was up-regulated in the HF steers following the ZG dietary phase. The *CSRP3* gene is involved in regulatory processes important for development and cellular differentiation and has been shown to function as a positive regulator of myogenesis^47^. Similarly, *SPX* has been shown to promote the proliferation and differentiation of skeletal muscle tissue^48^, whilst *PAMR1* has been shown to function in the regeneration of skeletal muscle. Moreover, myosin genes also displayed greater expression in both HF and CH L-RFI steers. Specifically these included, *MYH6* and *MYL6B*, both of which were up-regulated in the L-RFI HF steers following H1 dietary phase and *MYH4* and *MYH6*, both up-regulated in CH L-RFI steers following the ZG diet. Indeed, both *MYH6* and *MYL6B* were also previously identified as up-regulated in L-RFI cattle across tissues including anterior pituitary, skeletal muscle, liver, adipose and duodenum in the data of Weber et al.^25^. Additionally, the actin gene, *ACTC1* was also up-regulated in L-RFI steers (HF, ZG dietary phase). In skeletal muscle tissue, actin and myosin filaments combine to form myofibrils, which are organised into sacromeres functioning as the fundamental unit of muscle contraction. Although muscle fibre number is fixed at birth, the greater expression of these genes in the L-RFI steers may suggest greater hypertrophy in the L-RFI steers during later stages of life. Alternatively, the differential expression of such myofibre related genes may indicate towards a difference in the muscle fibre type between cattle divergent for RFI and which ultimately may contribute to the variation in energy requirements between L- and H-RFI cattle. Overall, results suggest a greater potential for muscle growth in the L-RFI steers across both breeds examined.

## Conclusion

Results from this study show that across two contrasting breed types and three diets varying in feed intake characteristics, chemical composition and nutritive value, no one common gene was determined as a key regulator or contributor to variation in RFI in skeletal muscle tissue. However, despite this within each comparison evaluated in the current study, genes with an associated function in fatty acid metabolism were differentially expressed between H- and L-RFI cattle, highlighting a clear role for such processes towards RFI divergence. Moreover, results from this study also highlight genes involved in greater skeletal muscle growth in L-RFI cattle. Overall results from this study identify common biological processes contributing to RFI across varying breed and dietary source types, however the lack of commonality of individual protein coding genes towards variation in RFI both within the current study and compared to the published literature, suggests other genomic features warrant evaluation in relation to RFI, for example those involved in transcriptional regulation and epigenetic functions to further unravel the underlying biological complexity of the RFI trait.

## Methods

### Animal model

This study was conducted at the Teagasc Animal and Grassland Research and Innovation Centre. All procedures involving animals were approved by the Teagasc Animal Ethics Committee and all procedures involving animals in the current study were conducted under an experimental license (AE19132/P029) from the Health Products Regulatory Authority in accordance with the cruelty to Animals Act 1876 and the European Communities (Amendment of Cruelty to Animals Act 1876) Regulations 2002 and 2005. All experiments were performed in accordance with relevant regulations and the ARRIVE (Animal Research: Reporting on In Vivo Experiments) guideline.

This experiment was conducted as part of a larger study designed to examine the within-animal repeatability of intake, growth and feed efficiency between the growing and finishing stages of the lifespan of CH and HF beef steers offered contrasting diets over consecutive test periods^19,20^. Briefly, one hundred and sixty seven cattle comprised of 90 CH and 77 HF were used in the study. Dietary feeding phases were as follows: (i) a high concentrate diet during the growing phase (H1); (ii) zero-grazed grass diet during the growing phase (ZG) and; (iii) high concentrate diet during the finishing phase (H2). Mean age and body weight for CH and HF at the start of the first high-concentrate phase were 283 d (SD = 18.3) and 394 kg (SD = 37.5), and 307 d (SD = 7.7) and 294 kg (SD = 41.8), respectively. The zero-grazed grass phase commenced 152 days, and the second high-concentrate phase commenced 371 days, after this. Mean body weight for CH and HF at the start of zero-grazed phase and second high-concentrate phase was 516 kg (SD = 37.3) and 440 kg (SD = 41.8), and 676 kg (SD = 50.0) and 611 kg (SD = 49.1), respectively. Following a dietary adaptation period (14 days), individual DMI and growth were measured over the three 70 d feeding phases, using a Calan gate system. During the high concentrate phases (H1 and H2), all steers received the same high-concentrate diet ad libitum (860 g/kg rolled barley, 60 g/kg soya bean meal, 60 g/kg molasses, and 20 g/kg minerals and vitamins) with a restricted allowance of grass silage daily. Throughout the interim ZG phase, all steers were individually offered zero-grazed grass (DM 183 g/kg) ad libitum. The grass herbage was harvested twice daily from Lolium perenne dominant swards using a zero-grazer. Chemical composition of diets offered is detailed in full in Higgins et al.^23^. Body-weight of all steers was measured, prior to feeding, at the beginning and end as well as at 14-day intervals throughout each dietary phase.

### Computation of RFI

Full details related to the computation of RFI trait are described in full in Higgins et al.^23^. Briefly, the residuals of the regression of dry matter intake on ADG, mid-test MBW within each breed, were used to compute individual RFI coefficients for each feeding phase using the GLM procedure of SAS 9.1 (SAS Inst. INC., Cary, NC). Residual feed intake was calculated for each animal as the difference between actual DMI and expected DMI. Animals were ranked within breed and feeding phase for H-RFI (n = 12) and L-RFI (n = 12).

### Tissue sample collection

*M. longissimus thoracis et lumborum* biopsies were harvested from animals deemed H- and L-RFI under local anaesthetic (5 mL Adrenacaine, Norbrook Laboratories (Ireland) Ltd.) as described by Keogh et al.^49^ at the end of each RFI measurement period. All surgical instruments used for tissue collection were sterilised and treated with 70% ethanol and RNaseZap (Ambion, Applera Ireland, Dublin, Ireland). Tissue biopsies were snap frozen in liquid nitrogen directly after collection. All samples were subsequently stored at − 80 °C pending further processing.

### RNA isolation and purification

Total RNA was isolated from 50 mg of muscle biopsy. Tissue samples were homogenised in 3 mL of QIAzol reagent using a rotor-strator tissue lyser (Qiagen, UK) and chloroform (Sigma-Aldrich Ireland, Dublin, Ireland). RNA was subsequently precipitated and purified using the RNeasy plus Universal kit (Qiagen, UK) according to the manufacturer’s guidelines, which included a step to remove any contaminating genomic DNA. The quantity of the RNA isolated was determined by measuring the absorbance at 260 nm using a Nanodrop spectrophotometer ND-1000 (Nanodrop Technologies, Wilmington, DE, USA). RNA quality was assessed on the Agilent Bioanalyser 2100 using the RNA 6000 Nano Lab Chip kit (Agilent Technologies Ireland Ltd., Dublin, Ireland). RNA quality was also verified by ensuring all RNA samples had an absorbance (A260/280) of between 1.8 to 2.0 and RIN (RNA Integrity Number) values of between 8 and 10 were deemed high quality. Any samples that had an (A260/280) absorbance of less than 1.8 were cleaned using a Zymo Research RNA clean & concentrator kit (Cambridge Biosciences, UK). All samples were deemed to be of sufficient high quality for subsequent cDNA synthesis.

### cDNA library preparation and sequencing

cDNA libraries were prepared from high quality RNA following the manufacturer’s instructions using the Illumina TruSeq stranded mRNA sample prep kit (Illumina, San Diego, CA, USA). For each sample, 1 μg of RNA was used for cDNA library preparation. Briefly, mRNA was purified from total RNA and subsequently fragmented. First strand cDNA was synthesised using Superscript II Reverse Transcriptase (Applied Biosystems Ltd., Life Technologies) followed by second strand synthesis using components of the Illumina TruSeq RNA sample prep kit. Adaptors were ligated to the cDNA which was subsequently enriched by 15 cycles of PCR. Libraries were validated on the Agilent Bioanalyser 2100 using the DNA 1000 Nano Lab Chip kit. cDNA concentration was assessed using Nanordrop spectrophotometer ND-1000 (Nanodrop Technologies, Wilmington, DE, USA) and samples with > 25 ng/μl were deemed suitable for further analysis. Following quality control procedures, individual RNA-seq libraries were pooled based on their respective sample-specific 6 bp adaptors and sequenced at 50 bp/sequence single-end read using an Illumina HiSeq 2500 sequencer. Approximately 22 million sequences per sample were generated.

### RNA-sequencing data analysis

FASTQC software (v0.11.5) was first used to check the quality of the raw sequencing reads. Cutadapt software (version 1.18.8) was then used to remove sequencing indexing adapters as well as any low quality reads. Trimmed input reads were then aligned to the bovine reference genome (ARS-UCD1.2) using Spliced Transcripts Alignment to a Reference (STAR) (v2.5.1). The STAR parameter “quantMode GeneCounts” was used to calculate the number of sequenced fragments overlapping all protein-coding genes from the ENSEMBLv87 annotation of the bovine genome. The number of counts of reads mapping to each annotated gene from STAR was then collated into a single file and used for subsequent differential gene expression.

Differentially expressed genes between each breed and dietary phase comparison were determining using the Bioconductor package, EdgeR (v3.20.9). Read counts were firstly filtered for lowly expressed genes through the removal of any gene with less than one count per million in at least half the number of samples across each comparison. Data were then normalised using the trimmed mean of M-value normalisation method, with exact tests then used for the detection of differentially expressed genes between H- and L-RFI steers across each breed and dietary phase comparison. Additionally, a separate analysis of all data was undertaken to evaluate the interaction of breed and diet, fitting RFI as a class variable and animal as a random effect within the model. Genes with a Benjamini–Hochberg false discovery rate of < 0.1 and a fold-change greater than 1.5 were considered differentially expressed. The resultant list of differentially expressed genes was then submitted to Ingenuity pathway analysis (Qiagen)^50^ in order to assign biological annotation and undertake biological pathway analysis.

### Ethics declaration

This study was conducted at the Teagasc Animal and Grassland Research and Innovation Centre. All procedures involving animals were approved by the Teagasc Animal Ethics Committee and all procedures involving animals in the current study were conducted under an experimental license (AE19132/P029) from the Health Products Regulatory Authority in accordance with the cruelty to Animals Act 1876 and the European Communities (Amendment of Cruelty to Animals Act 1876) Regulations 2002 and 2005. All experiments were performed in accordance with relevant regulations and the ARRIVE (Animal Research: Reporting on In Vivo Experiments) guideline.

## Supplementary Information

Supplementary Table 1.

Supplementary Table 2.

Supplementary Table 3.

## Data Availability

The sequencing data underlying this article are available in NCBI’s Gene Expression Omnibus at [https://www.ncbi.nlm.nih.gov/geo/] and can be accessed with unique GEO ID GSE113135.
